# Supplementary material for: Genome-wide association study identifies novel type II diabetes risk loci in Jordan subpopulations
Source: PeerJ. 2017 Aug 17;5:e3618. doi: 10.7717/peerj.3618 (PMC5563445; doi:10.7717/peerj.3618)
Supplement: Supplemental Information 1 [file peerj-05-3618-s001.pdf]

Genome-Wide Association Study Identifies Novel Type II Diabetes Risk Loci in Jordan Subpopulations

Rana Dajani, Jin Li, Zhi Wei, Michael E. March, Qianghua Xia, Yousef Khader, Nancy Hakooz, Raja Fatahallah, Mohammed El-Khateeb, Ala Arafat, Tareq Saleh, Abdel Rahman Dajani, Zaid Al-Abbadi, Mohamed Abdul Qader, Abdel Halim Shiyab, Anwar Bateiha, Kamel Ajlouni, Hakon Hakonarson

Supplementary Information

Supplementary Figures

Supplementary Figure 1. Manhattan plot of the result from the meta-analysis of Chechen and Circassian groups.

SNPs are sorted by chromosomal location along X axis against their -log10(*P*-value) shown on Y axis. The horizontal line represents the genome-wide significance level $p=5x10^{-8}$.

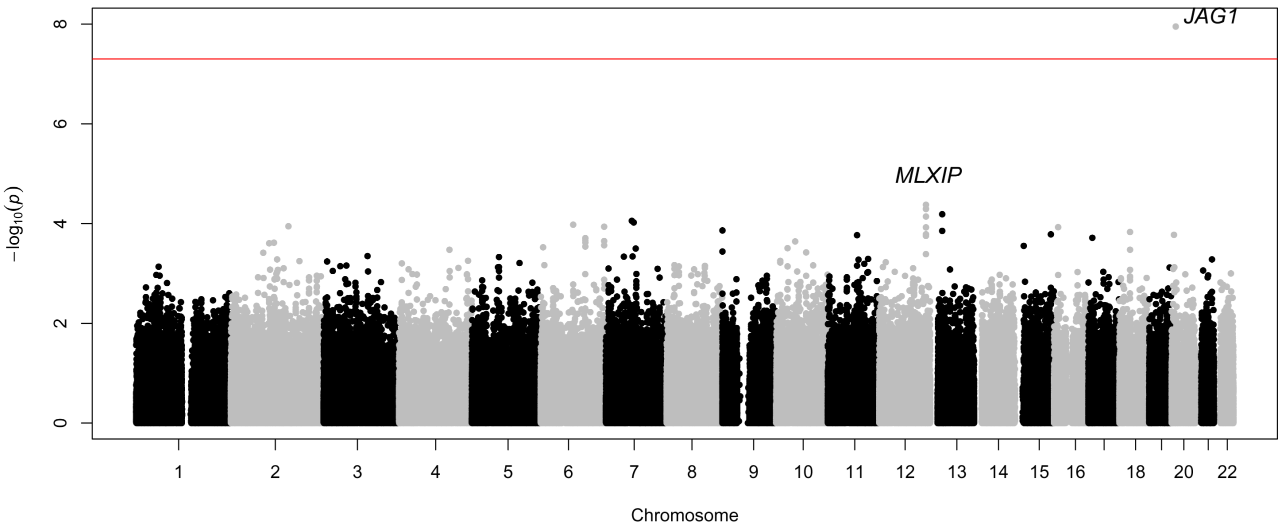

Supplementary Figure 2: The association between SNP rs6134031 genotype and gene *JAG1* expression level in esophagus muscularis and pancreas.

The relationship between SNP rs6134031genotype and gene *JAG1* expression level was evaluated in GTEx Portal (GTEx Consortium 2015). Their correlation in **(a)** Esophagus muscularis and **(b)** Pancreas are shown. The different genotype groups of SNP rs6134031 are indicated on the X-axis, with C being the reference allele and T being the alternative allele. The Rank Normalized gene expression level of gene *JAG1* is shown on the Y-axis. The black line in the box plot denotes the median expression level in each genotype group. The bottom and the top border of each box represent the first quartile and the third quartile of the rank normalized gene expression level, respectively. The end of the lower whisker and that of the upper whisker represent the 1.5 interquartile range (IQR). The small grey circles denote *JAG1* expression level of each individual.

a **b**

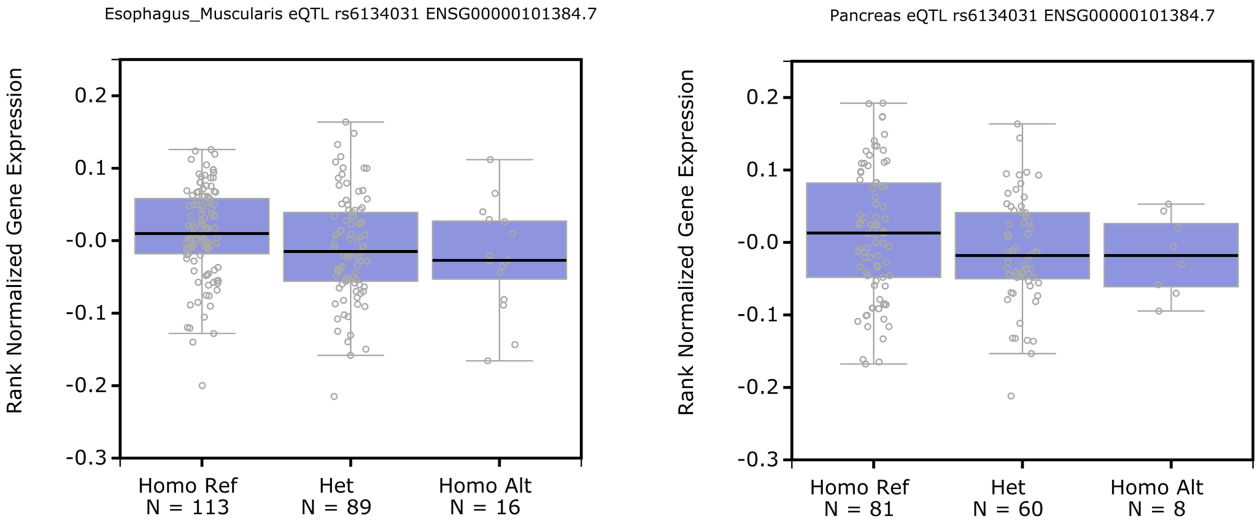

Beta=-0.15, P=0.0073 Beta=-0.13, P=0.071

Supplementary Figure 3. Epigenetics profiles of the immediate genomic region of cg22729539.

(a) DNase hypersensitivity peaks in pancreatic islets; **(b)** chromatin states in pancreatic islets and human liver carcinoma cells HepG2; **(c)** histone markers in adult liver and pancreatic islets; **(d)** Transcription factor binding sites in pancreatic islets and pancreatic progenitor cells.

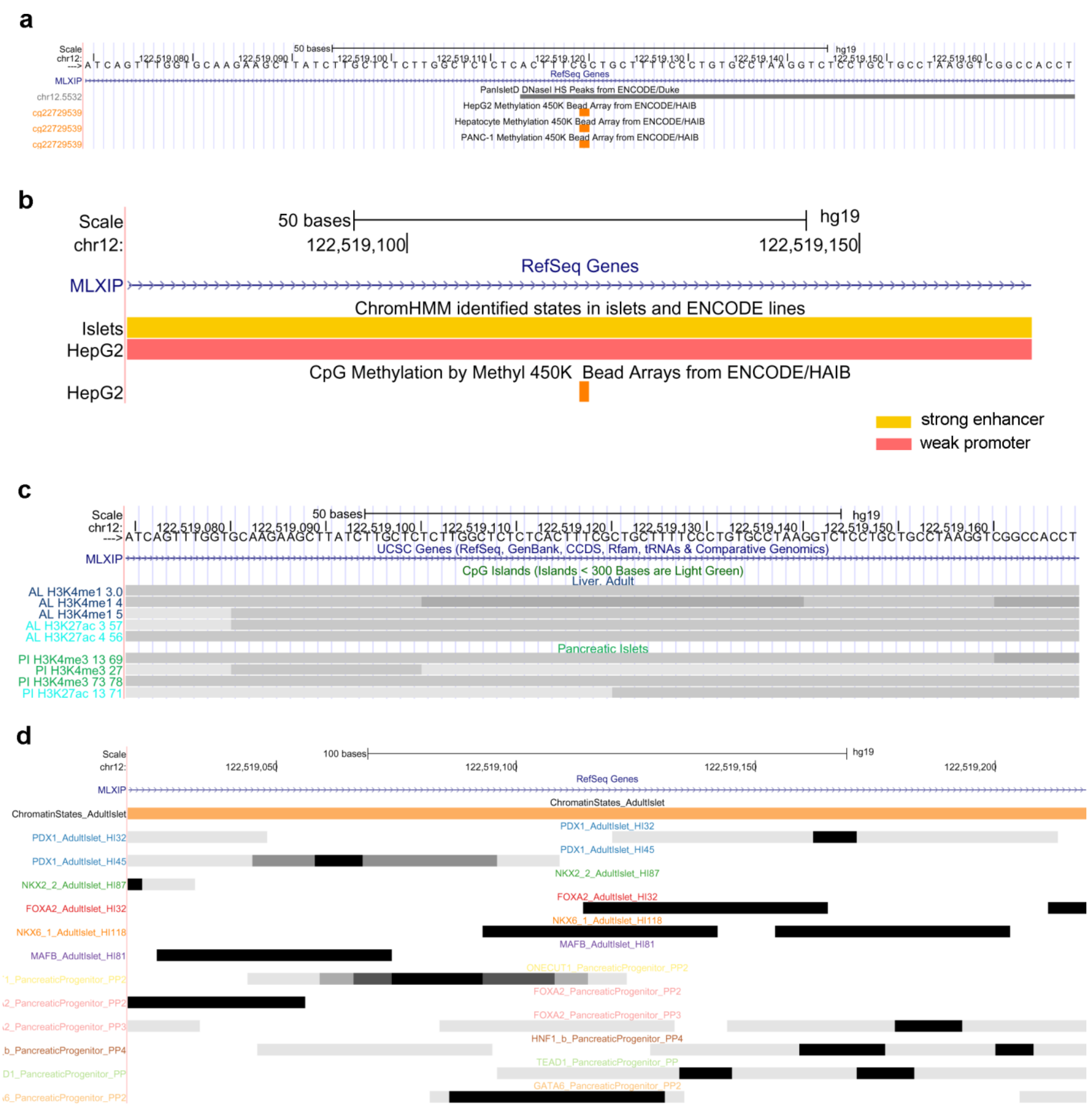

Supplementary Figure 4: Box plot showing the expression level of *JAG1* gene in different human tissue/cell types, based on GTEx RNA-seq data.

The plot was generated via GTEx (GTEx Consortium 2015) online portal. Tissue/cell types are indicated on the X-axis, and the normalized gene expression levels are indicated by the RPKM (Reads Per Kilobase of transcript per Million mapped reads) values on the Y-axis. The dark black line in each box shows the median expression level in each cell/tissue type, and the bottom/top border of each box indicates the first/third quartile of the RPKM. The end of the lower whisker and that of the upper whisker represent the lowest and the highest datum within the 1.5 interquartile range (IQR) of the lower quartile and the upper quartile, respectively; and the grey circles represent data points outside of this range.

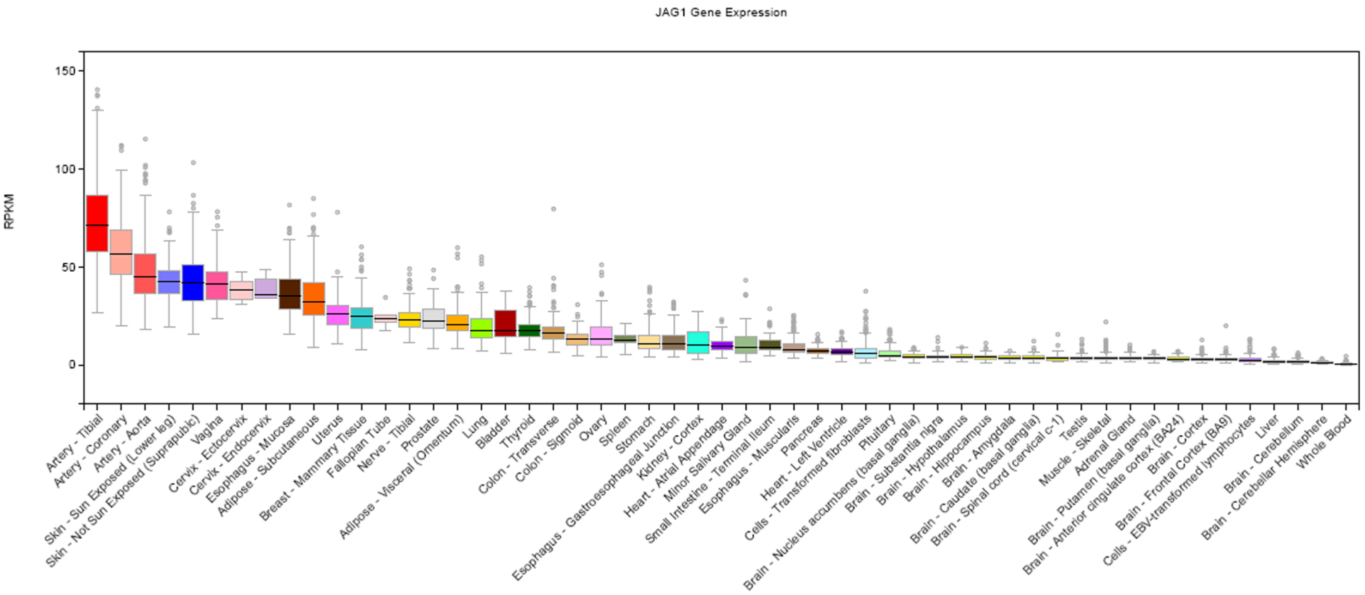

Supplementary Figure 5: Bar graph showing the expression level of *JAG1* gene in different human tissue/cell types, based on BioGPS microarray data.

The expression data were downloaded from BioGPS (Wu et al. 2016; Wu et al. 2009) web portal (http://biogps.org/#goto=welcome)

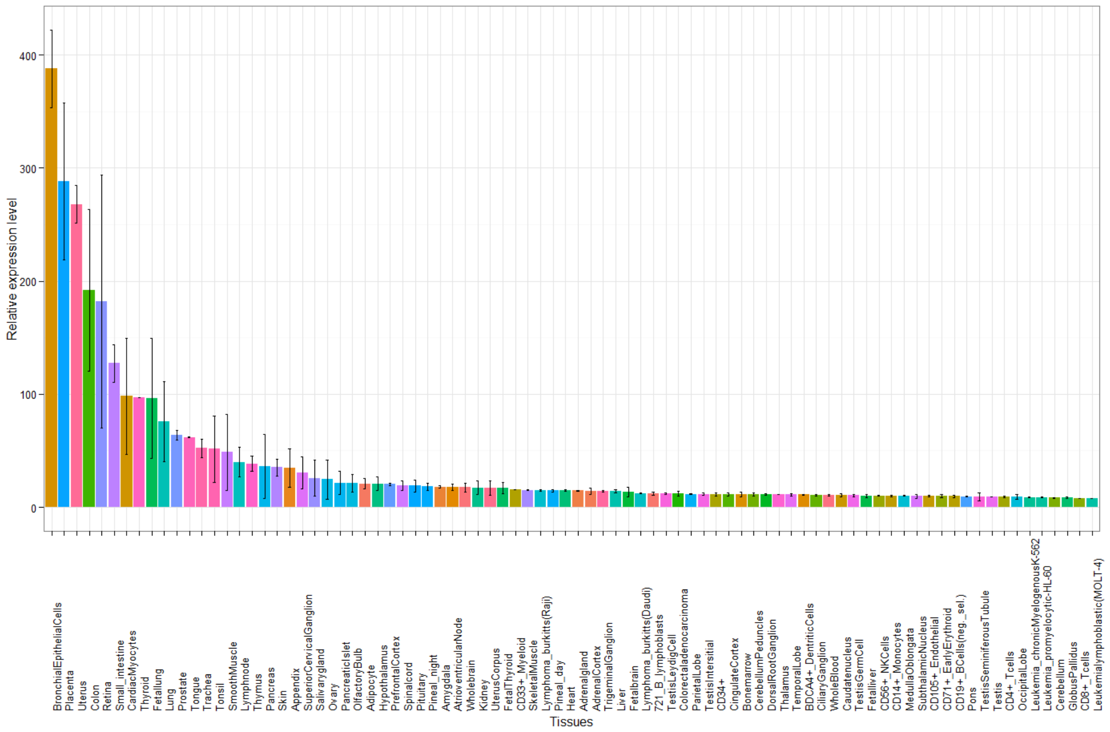

Supplementary Figure 6: Box plot showing the expression level of *MLXIP* gene in different human tissue/cell types, based on GTEx RNA-seq data.

The box plot was generated via GTEx (GTEx Consortium 2015) online portal. Tissue/cell types are indicated on the X-axis, and the normalized gene expression levels are indicated by the RPKM (Reads Per Kilobase of transcript per Million mapped reads) values on the Y-axis. The dark black line in each box shows the median expression level in each cell/tissue type, and the bottom/top border of each box indicates the first/third quartile of the RPKM. The end of the lower whisker and that of the upper whisker represent the lowest and the highest datum within the 1.5 interquartile range (IQR) of the lower quartile and the upper quartile, respectively; and the grey circles represent data points outside of this range.

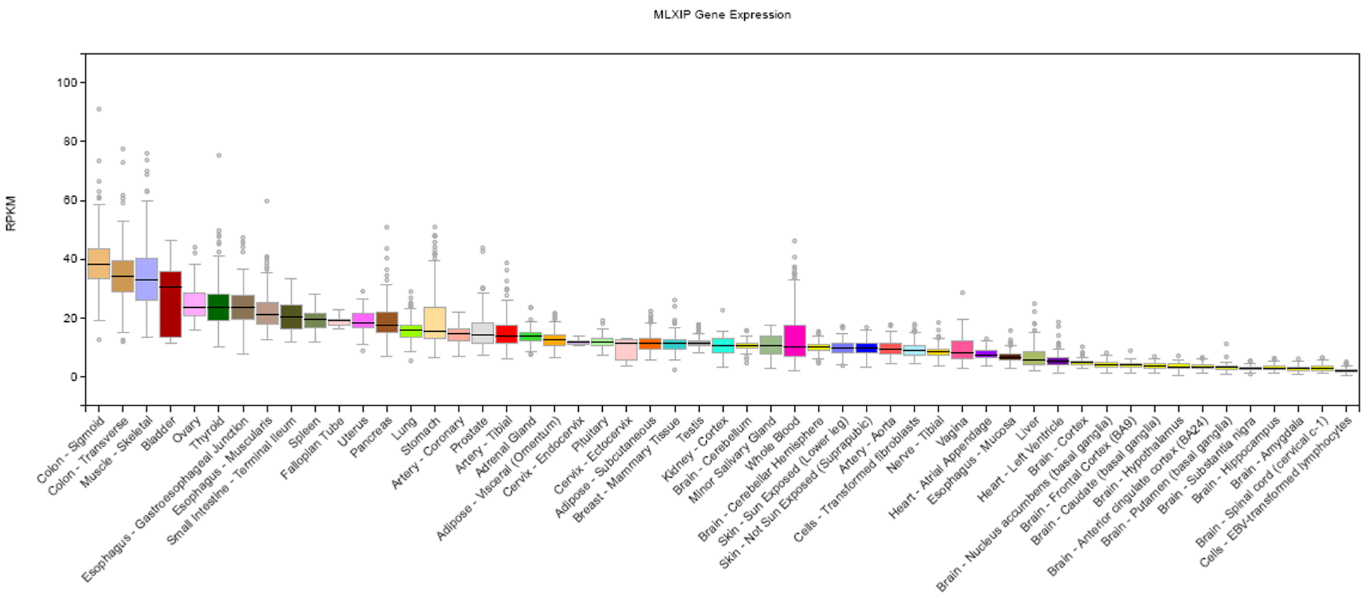

Supplementary Figure 7: Bar graph showing the expression level of *MLXIP* gene in different human tissue/cell types, based on BioGPS microarray data.

The expression data were downloaded from BioGPS (Wu et al. 2016; Wu et al. 2009) web portal (http://biogps.org/#goto=welcome)

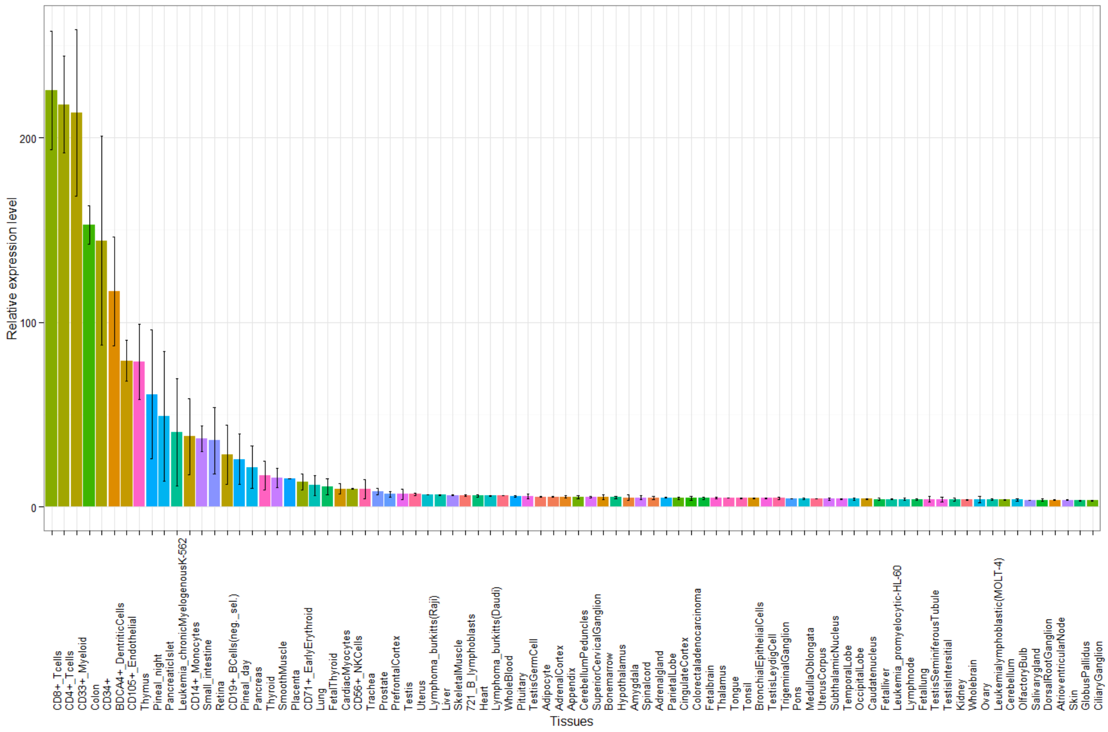

Supplementary Figure 8: Summary of *JAG1* knockout mice phenotype.

The blue boxes indicate the presence of the defects. Information was extracted from http://www.informatics.jax.org/marker/MGI:1095416 (Blake et al. 2017; Finger et al. 2017).

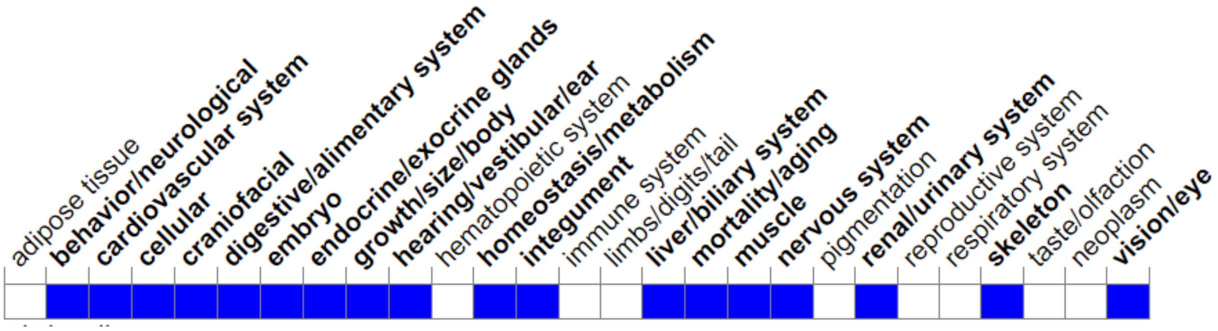

Supplementary Tables

Supplementary Table 1: Top associations from Type 2 Diabetes GWAS of Circassian subpopulation.

SNP	Chr	Pos(hg19)	Closest Gene	A1	OR (95% CI)	P-value
rs6134031	20	10752610	*JAG1*	T	9.48 (3.09,29.07)	8.36×10^{-5}
rs12403551	1	101864215	*RNU6-6*	A	4.09 (1.76,9.51)	1.06×10^{-3}
rs349488	5	62557698	*IPO11*	A	4.17 (1.77,9.80)	1.06×10^{-3}
rs11760752	7	73023062	*MLXIPL*	A	5.16 (1.92,13.89)	1.15×10^{-3}
rs11770924	7	122659240	*TAS2R16*	G	0.20 (0.07,0.53)	1.29×10^{-3}
rs11704261	22	33206863	*SYN3,TIMP3*	T	7.49 (2.19,25.69)	1.35×10^{-3}
rs7290713	22	33188545	*SYN3*	T	7.49 (2.19,25.69)	1.35×10^{-3}
rs2617892	18	28142539	*MIR302F*	C	2.74 (1.48,5.08)	1.36×10^{-3}
rs2433086	8	25530161	*CDCA2*	A	0.20 (0.08,0.54)	1.38×10^{-3}
rs10486867	7	70071920	*AUTS2*	A	0.27 (0.12,0.61)	1.54×10^{-3}

SNP – single nucleotide polymorphism; Chr – chromosome; Pos – Position; A1 – minor allele; OR – odds ratio; CI – confidence interval

Supplementary Table 2: Top associations from Type 2 Diabetes GWAS of Chechen subpopulation.

SNP	Chr	Pos(hg19)	Closest Gene	A1	OR (95% CI)	P-value
rs6134031	20	10752610	*JAG1*	T	9.84 (3.33,29.02)	$3.45x10^{-5}$
rs4398502	4	174819200	*FBXO8*	G	0.14 (0.06,0.36)	$3.87x10^{-5}$
rs12283277	11	80399178	*intergenic*	C	5.96 (2.55,13.96)	$3.88x10^{-5}$
rs203035	17	50069517	*CA10*	A	6.55 (2.67,16.08)	$4.07x10^{-5}$
rs203038	17	50070614	*CA10*	T	6.55 (2.67,16.08)	$4.07x10^{-5}$
rs188028	17	49991836	*CA10*	A	6.94 (2.73,17.64)	$4.71x10^{-5}$
rs203049	17	50078989	*CA10*	A	6.16 (2.56,14.82)	$4.96x10^{-5}$
rs12617659	2	121309759	*LOC84931*	T	11.43 (3.51,37.18)	$5.17x10^{-5}$
rs7264874	20	56045814	*CTCFL*	T	14.65 (3.99,53.82)	$5.27x10^{-5}$
rs1945378	11	80429527	*intergenic*	G	6.08 (2.53,14.57)	$5.28x10^{-5}$

SNP – single nucleotide polymorphism; Chr – chromosome; Pos – Position; A1 – minor allele; OR – odds ratio; CI – confidence interval

Supplementary Table 3: The histone marks, DNAse hypersensitive sites near cg22729539 (chr12:122081214-122081214, hg38).

Only cell lines which are biologically relevant to Type II Diabetes are shown. Data were extracted via Haploreg (Ward & Kellis 2012), based on Roadmap (Leung et al. 2015) and ENCODE datasets (Encode Project Consortium 2012).

Chr	Pos (hg38)	H3K4me1	H3K4me3	H3K27ac	H3K9ac	DNase	Proteins bound
12	122080733	E025,E063,E066,E087,E098,E107,E108,E120,E121	E025,E063,E066,E098,E107,E108,E120,E121	E063,E066,E087,E098,E108,E120,E121	E025,E063,E066,E087,E107,E108,E120,E121	E098,E120,E121	CJUN, POL2, P300, RAD21
12	122080829	E025,E063,E066,E087,E098,E107,E108,E120,E121	E025,E063,E066,E098,E107,E108,E120,E121	E063,E066,E087,E098,E108,E120,E121	E025,E063,E066,E087,E107,E108,E120,E121	E121	POL2
12	122080943	E025,E063,E066,E087,E098,E107,E108,E120,E121	E025,E063,E066,E098,E107,E108,E120,E121	E063,E066,E087,E098,E108,E120,E121	E025,E063,E066,E087,E107,E108,E120,E121	E098	
12	122081053	E025,E063,E066,E087,E098,E107,E108,E120,E121	E025,E063,E066,E087,E098,E107,E108,E120,E121	E063,E066,E087,E098,E108,E120,E121	E025,E063,E066,E087,E107,E108,E120,E121		CEBPB
12	122081110	E025,E063,E066,E087,E098,E107,E108,E120,E121	E025,E063,E066,E087,E098,E107,E108,E120,E121	E063,E066,E087,E098,E108,E120,E121	E025,E063,E066,E087,E107,E108,E120,E121	E098,E121	CEBPB
12	122081119	E025,E063,E066,E087,E098,E107,E108,E120,E121	E025,E063,E066,E087,E098,E107,E108,E120,E121	E063,E066,E087,E098,E108,E120,E121	E025,E063,E066,E087,E107,E108,E120,E121	E098,E120,E121	CEBPB
12	122081164	E025,E063,E066,E087,E098,E107,E108,E120,E121	E025,E063,E066,E087,E098,E107,E108,E120,E121	E063,E066,E087,E098,E108,E120,E121	E025,E063,E066,E087,E107,E108,E120,E121	E098,E120,E121	CEBPB
12	122081182	E025,E063,E066,E087,E098,E107,E108,E120,E121	E025,E063,E066,E087,E098,E107,E108,E120,E121	E063,E066,E087,E098,E108,E120,E121	E025,E063,E066,E087,E107,E108,E120,E121	E098,E120,E121	CEBPB
12	122081287	E025,E063,E066,E087,E098,E107,E108,E120,E121	E025,E063,E066,E087,E098,E107,E108,E120,E121	E063,E066,E087,E098,E108,E120,E121	E025,E063,E066,E087,E107,E108,E120,E121	E120,E121	
12	122081427	E025,E063,E066,E087,E098,E107,E108,E120,E121	E025,E063,E066,E087,E098,E107,E108,E120,E121	E063,E066,E087,E098,E108,E120,E121	E025,E063,E066,E087,E107,E108,E120,E121	E098,E120,E121	
12	122081486	E025,E063,E066,E087,E098,E107,E108,E120,	E025,E063,E066,E087,E098,E107,E108,E	E063,E066,E087,E098,E108,E120,E121	E025,E063,E066,E087,E107,E108,E12		

		E121	120,E121		0,E121
12	122081564	E025,E063,E066,E087, E098,E107,E108,E120, E121	E025,E063,E066,E08 7,E098,E107,E108,E 120,E121	E063,E066,E087,E0 98,E108,E120,E121	E025,E063,E066,E 087,E107,E108,E12 0,E121
12	122081573	E025,E063,E066,E087, E098,E107,E108,E120, E121	E025,E063,E066,E08 7,E098,E107,E108,E 120,E121	E063,E066,E087,E0 98,E108,E120,E121	E025,E063,E066,E 087,E107,E108,E12 0,E121

Cell line description: E025= Adipose Derived Mesenchymal Stem Cell Cultured Cells; E063= Adipose Nuclei; E066=Liver; E087= Pancreatic Islets; E098= Pancreas; E107= Skeletal Muscle Male; E108= Skeletal Muscle Female; E120= HSMM Skeletal Muscle Myoblasts Cells; E121= HSMM cell derived Skeletal Muscle Myotubes Cells.

Supplementary Table 4: The association statistics of genes that have been reported to be associated with T2D in previous genetic or genomic studies.

Nominally significant associations are highlighted.

Gene	SNP	Chr	Pos (hg19)	A1	OR	P
FAF1	rs17387024	1	50993178	C	2.19	**5.26×10^{-3}**
TGFBR3	rs17881430	1	92327707	A	1.82	**4.56×10^{-2}**
F3	rs17111996	1	95122518	T	0.63	**4.99×10^{-2}**
SLC44A3	rs735937	1	95335769	C	0.58	**3.80×10^{-2}**
ADAM30	rs10923918	1	120412885	T	0.59	9.57×10^{-2}
NOTCH2	rs17258579	1	120469077	C	1.63	0.20
CR2	rs2182911	1	207660071	C	1.78	6.97×10^{-2}
PROX1	rs3767848	1	214173840	A	1.72	**3.51×10^{-2}**
LYPLAL1	rs12123734	1	219577622	A	1.65	**3.51×10^{-2}**
DUSP5P,RHOU	rs12407568	1	228783329	T	1.92	0.55
RHOU	rs496663	1	229098232	C	1.80	**1.95×10^{-2}**
PCNXL2	rs6693854	1	233215885	C	0.48	**2.77×10^{-2}**
TMEM18	rs4241319	2	603079	G	1.91	**1.28×10^{-2}**
THADA	rs1549723	2	43460514	A	0.58	9.24×10^{-2}
BCL11A	rs356993	2	60750560	T	1.46	0.15
TMEM163	rs4953921	2	135286448	G	1.40	0.17
RND3	rs193046	2	150904778	C	7.35	**1.13×10^{-4}**
RBM43	rs11675356	2	151931005	A	0.55	5.71×10^{-2}
ITGB6	rs10202454	2	161044155	G	4.19	**3.04×10^{-2}**
RBMS1	rs532146	2	161622892	G	2.12	**2.65×10^{-2}**
GRB14	rs12474679	2	165346592	G	1.66	5.70×10^{-2}
IRS1	rs16822601	2	227616748	C	2.63	6.28×10^{-2}
DNER	rs977041	2	230350848	A	1.68	**3.64×10^{-2}**
SYN2	rs4684093	3	12025330	C	1.56	0.10
SYN2,TIMP4	rs17035943	3	12194584	C	0.21	0.16
PPARG	rs12485478	3	12351223	G	5.66	7.00×10^{-2}
UBE2E2	rs13096681	3	22686512	A	2.70	**6.00×10^{-3}**
MIR548AC,UBE2E2	rs1353322	3	23390301	G	0.61	5.67×10^{-2}
PSMD6-AS2	rs4688181	3	63989456	G	9.40	**9.30×10^{-3}**
PSMD6,PSMD6-AS2	rs2280162	3	63997491	A	1.92	5.63×10^{-2}
PSMD6	rs166229	3	64021228	G	1.27	0.37
ADAMTS9	rs9871177	3	64480785	C	0.29	**1.60×10^{-3}**
ADAMTS9-AS2,MIR548A2	rs318725	3	64755337	G	0.62	**4.76×10^{-2}**
ADAMTS9-AS2	rs9790242	3	65103850	A	2.81	**1.91×10^{-2}**
ZPLD1	rs9854367	3	102259425	C	1.90	**3.75×10^{-2}**
ADCY5	rs4678005	3	123021298	C	1.78	**3.20×10^{-2}**
PLS1	rs1983434	3	142347757	G	0.67	0.12

PEX5L	rs2003004	3	179836036	G	2.33	**$1.27x10^{-2}$**
C3orf65,IGF2BP2	rs17826758	3	185431283	G	0.96	0.91
IGF2BP2	rs12639517	3	185574131	T	1.43	0.16
ST6GAL1	rs10513809	3	186667250	T	0.19	$5.28x10^{-2}$
LPP-AS2	rs7651169	3	187814477	G	1.94	**$1.51x10^{-2}$**
FLJ42393,LPP	rs4577458	3	187897424	C	0.69	0.15
LPP	rs2278096	3	188590650	T	2.30	**$9.35x10^{-3}$**
MAEA	rs3817604	4	1291337	T	0.61	0.31
WFS1	rs10002743	4	6276581	G	0.51	**$4.73x10^{-2}$**
PPP2R2C	rs7377185	4	6407801	T	2.03	**$8.57x10^{-3}$**
COX7B2	rs9291290	4	46878105	T	0.56	**$3.08x10^{-2}$**
GABRA4	rs2055940	4	46997913	A	2.47	**$1.05x10^{-3}$**
TMEM154	rs6849505	4	153573491	T	0.57	0.25
ANP32C,MARCH1	rs2288675	4	165118652	T	1.30	0.27
ARL15	rs154021	5	53463117	C	1.85	**$3.03x10^{-2}$**
MAP3K1	rs3736430	5	56180480	G	2.69	**$2.09x10^{-2}$**
ZBED3	rs2914141	5	76378192	T	1.58	0.12
ZBED3-AS1	rs7708285	5	76425867	G	1.80	**$4.80x10^{-2}$**
CETN3	rs16868525	5	89640426	A	4.42	**$1.36x10^{-2}$**
ZNF608	rs7705693	5	124084365	T	1.94	**$1.25x10^{-2}$**
PCBD2	rs9285933	5	134257519	A	0.43	$5.24x10^{-2}$
RREB1	rs7757080	6	6929780	C	2.77	**$1.67x10^{-2}$**
SSR1	rs9968917	6	7267002	A	0.46	0.25
CDKAL1	rs16884481	6	21076048	T	3.06	**$1.31x10^{-2}$**
TCF19	rs1044870	6	31130821	T	0.54	0.20
POU5F1	rs9263796	6	31132883	T	0.54	0.20
HLA-DQA2	rs3957146	6	32681530	C	3.20	**$1.97x10^{-3}$**
PPARD	rs4713854	6	35381099	C	2.79	$5.39x10^{-2}$
ZFAND3	rs12529345	6	37755453	T	2.21	**$3.16x10^{-3}$**
KCNK16	rs11758206	6	39296416	T	3.08	0.22
VEGFA	rs4714696	6	43719993	C	1.63	$7.78x10^{-2}$
C6orf57	rs9455167	6	71291087	G	0.06	0.11
RPS12,SNORD100	rs9389034	6	133138010	C	1.01	0.97
RPS12	rs12193474	6	133186168	C	0.54	0.18
IL20RA	rs276550	6	137401777	C	0.61	$6.92x10^{-2}$
SASH1	rs6570846	6	148682423	A	2.11	**$3.80x10^{-3}$**
ETV1	rs9792016	7	13857235	G	2.53	**$1.89x10^{-3}$**
DGKB	rs10487790	7	15042907	C	2.22	**$2.08x10^{-2}$**
AGR2	rs11773294	7	16839835	C	2.13	**$3.29x10^{-2}$**
AGR3	rs3944100	7	17054346	C	1.92	**$1.76x10^{-2}$**
JAZF1	rs10258887	7	27940578	C	3.67	**$1.85x10^{-2}$**
JAZF1-AS1	rs4722772	7	28285903	C	1.52	0.14
CRHR2	rs255125	7	30743011	A	0.70	0.23
ELMO1	rs10488653	7	37491704	C	3.22	**$2.04x10^{-2}$**

ACHE	rs6980219	7	100542445	T	0.67	0.13
GCC1	rs17714284	7	127147205	A	2.39	$5.28x10^{-2}$
PAX4	rs327510	7	127257894	G	1.26	0.40
SND1,SND1-IT1	rs1362897	7	127638703	A	1.28	0.43
SND1	rs6467157	7	127660763	C	0.64	0.17
LRRC4,SND1	rs6944446	7	127667516	A	0.84	0.50
LEP	rs1116656	7	127910047	A	1.64	$5.64x10^{-2}$
KLF14	rs1364422	7	130445981	T	1.72	$6.04x10^{-2}$
GFRA2	rs17060577	8	21673762	G	0.38	**$1.73x10^{-2}$**
DOK2	rs11780601	8	21717841	T	1.84	**$4.78x10^{-2}$**
ANK1	rs12549902	8	41509259	G	0.55	**$1.83x10^{-2}$**
TP53INP1	rs493506	8	95965135	G	2.01	0.12
SLC30A8	rs7828267	8	118236041	C	2.20	**$1.34x10^{-2}$**
WISP1	rs13256058	8	134195513	A	1.54	$8.47x10^{-2}$
GLIS3	rs10973736	9	3838888	T	2.43	**$7.17x10^{-3}$**
GLIS3,GLIS3-AS1	rs10974067	9	3900982	C	1.14	0.65
PTPRD	rs10441691	9	8125336	A	2.48	**$6.65x10^{-3}$**
CDKN2A	rs3731217	9	21984661	C	1.41	0.30
CDKN2B,CDKN2B-AS1	rs1063192	9	22003367	G	0.83	0.48
CDKN2B-AS1	rs10965267	9	22161828	G	0.39	$6.77x10^{-2}$
LINGO2	rs12347591	9	28342014	C	2.37	**$6.15x10^{-3}$**
TLE4	rs10123659	9	83235735	G	0.35	**$1.37x10^{-2}$**
TLE1	rs10491932	9	83333000	A	0.40	**$1.44x10^{-2}$**
SYK	rs10993708	9	93606091	A	45.95	**$2.62x10^{-2}$**
AUH	rs7025567	9	93969769	A	1.54	0.23
PALM2	rs10979994	9	112438399	T	2.59	**$6.75x10^{-3}$**
PALM2-AKAP2	rs7030564	9	112778952	C	1.46	0.15
AKAP2,PALM2-AKAP2	rs496709	9	112879944	G	0.63	0.16
SVEP1	rs17733560	9	113124562	C	0.43	**$2.42x10^{-2}$**
MUSK	rs10980575	9	113585503	A	2.09	**$3.37x10^{-2}$**
GPSM1	rs28380074	9	139225139	G	0.64	$6.24x10^{-2}$
CDC123	rs7916879	10	12300790	A	1.62	$6.29x10^{-2}$
CAMK1D	rs7899150	10	12509131	T	1.93	**$2.68x10^{-2}$**
VPS26A	rs12256853	10	70930360	G	1.52	0.25
C10orf35	rs12569387	10	71460661	T	1.55	0.12
ZMIZ1	rs10824715	10	80858151	C	1.74	**$2.77x10^{-2}$**
PTEN	rs7079271	10	89833307	G	1.92	**$2.02x10^{-2}$**
IDE	rs11187061	10	94305409	T	1.65	$6.10x10^{-2}$
KIF11	rs12256435	10	94353278	T	2.35	**$4.19x10^{-2}$**
HHEX	rs2497313	10	94480391	T	1.85	$9.71x10^{-2}$
TCF7L2	rs12098403	10	115006062	A	2.94	**$2.31x10^{-2}$**
GRK5	rs11198929	10	121207272	T	0.56	**$3.37x10^{-2}$**
PLEKHA1	rs1998345	10	124124306	A	1.44	0.15
TCERG1L	rs4751353	10	133078257	A	2.07	**$1.25x10^{-2}$**

KCNQ1	rs12274567	11	2474956	A	2.39	**4.68x10^{-2}**
KCNQ1,KCNQ1OT1	rs10832514	11	2688526	A	1.49	0.11
GALNTL4	rs4441010	11	11269086	G	2.02	**1.38x10^{-2}**
CSNK2A1P,GALNTL4	rs2071460	11	11374269	G	1.12	0.63
KCNJ11	rs2074314	11	17411821	C	1.54	0.11
MTNR1B	rs6483208	11	92705844	G	5.47	**1.42x10^{-2}**
ATM	rs17412803	11	108217384	G	3.61	**4.29x10^{-2}**
BARX2	rs3019806	11	129374559	T	2.11	**2.13x10^{-2}**
TMEM45B	rs10894138	11	129657401	T	1.33	0.22
KLHDC5	rs11829884	12	27994433	A	4.06	7.93x10^{-2}
DCD	rs4759097	12	55075774	G	1.38	0.19
MSRB3	rs10878260	12	65724724	A	0.39	**5.81x10^{-3}**
HMGA2	rs1480468	12	66342117	A	1.84	9.06x10^{-2}
TSPAN8	rs10506627	12	71666552	C	4.00	0.18
LGR5	rs1298142	12	71941490	C	2.03	**3.26x10^{-2}**
HNF1A-AS1	rs11065374	12	121400205	C	1.89	**1.01x10^{-2}**
HNF1A	rs1169302	12	121432302	G	0.58	**3.16x10^{-2}**
MPHOSPH9	rs1727301	12	123664514	A	1.45	0.14
SGCG	rs17078439	13	23766378	C	2.30	**2.60x10^{-2}**
SACS	rs9578601	13	24018842	T	1.88	6.50x10^{-2}
RNF6	rs11619338	13	26745831	T	0.45	**2.55x10^{-2}**
SPRY2	rs9574595	13	80772435	T	0.43	5.73x10^{-2}
RASGRP1	rs4465567	15	38798246	C	0.50	**1.53x10^{-2}**
HMG20A	rs16953967	15	77836521	T	0.64	9.54x10^{-2}
ZFAND6	rs4778745	15	80363413	G	1.58	0.25
AP3S2,C15orf38-AP3S2	rs12440213	15	90366032	T	0.66	0.20
C15orf38-AP3S2	rs3803533	15	90441937	T	1.19	0.70
C15orf38,C15orf38-AP3S2	rs1702161	15	90480586	G	1.45	0.44
PRC1	rs4932182	15	91508803	A	1.13	0.61
LOC100507118,PRC1	rs8042680	15	91521337	A	1.19	0.45
TNRC6A	rs12103149	16	24673898	A	1.58	0.13
FTO	rs17236708	16	54128426	A	0.63	6.98x10^{-2}
CCDC102A	rs9922763	16	57546146	A	1.40	0.22
WWOX	rs7200433	16	78955140	T	3.12	**5.72x10^{-3}**
CMIP	rs4888159	16	81646894	T	2.09	**2.95x10^{-3}**
CMIP,LOC100129617	rs1128658	16	81700808	A	1.34	0.53
NXN	rs910922	17	710317	A	2.23	**1.46x10^{-2}**
SRR	rs12450028	17	2207425	T	1.74	**2.35x10^{-2}**
SLC16A13	rs11078663	17	6942108	C	0.84	0.49
SLC16A11	rs193399	17	6962272	A	2.24	0.27
HNF1B	rs17138522	17	36043734	G	4.61	**1.92x10^{-2}**
LPIN2	rs3826637	18	2937646	G	0.59	6.09x10^{-2}
LOC727896,LPIN2	rs643507	18	2946291	C	0.63	0.44
LAMA1	rs679668	18	7039943	A	2.74	**3.29x10^{-3}**

MC4R	rs11152221	18	58017249	T	1.95	**3.08x10^{-2}**
PEPD	rs891035	19	34060938	G	0.49	**1.94x10^{-2}**
GIPR	rs12976464	19	46162399	A	0.82	0.65
R3HDML	rs3746570	20	42965863	T	1.58	0.12
HNF4A	rs2425640	20	43028037	A	0.63	6.00x10^{-2}
MIR5095,RBM38	rs439749	20	55968113	G	0.64	0.10
CTCFL	rs7264874	20	56045814	T	4.53	**3.39x10^{-3}**
PCK1	rs1543375	20	56130620	G	1.67	**3.47x10^{-2}**
HUNK	rs2833579	21	33328529	G	1.53	8.47x10^{-2}
LIMK2	rs5997917	22	31621792	A	1.76	0.11
SULT4A1	rs470085	22	44219716	C	4.61	**1.95x10^{-2}**
FAM58A	rs4898361	X	152872445	A	2.33	5.11x10^{-2}
DUSP9	rs3761536	X	152906292	T	0.58	0.22

SNP – single nucleotide polymorphism; Chr – chromosome; Pos – Position; A1 – minor allele; OR – odds ratio

References:

Blake JA, Eppig JT, Kadin JA, Richardson JE, Smith CL, Bult CJ, and the Mouse Genome Database G. 2017. Mouse Genome Database (MGD)-2017: community knowledge resource for the laboratory mouse. *Nucleic Acids Res* 45:D723-D729. 10.1093/nar/gkw1040

Encode Project Consortium. 2012. An integrated encyclopedia of DNA elements in the human genome. *Nature* 489:57-74. 10.1038/nature11247

Finger JH, Smith CM, Hayamizu TF, McCright IJ, Xu J, Law M, Shaw DR, Baldarelli RM, Beal JS, Blodgett O, Campbell JW, Corbani LE, Lewis JR, Forthofer KL, Frost PJ, Giannatto SC, Hutchins LN, Miers DB, Motenko H, Stone KR, Eppig JT, Kadin JA, Richardson JE, and Ringwald M. 2017. The mouse Gene Expression Database (GXD): 2017 update. *Nucleic Acids Res* 45:D730-D736. 10.1093/nar/gkw1073

GTEx Consortium. 2015. Human genomics. The Genotype-Tissue Expression (GTEx) pilot analysis: multitissue gene regulation in humans. *Science* 348:648-660. 10.1126/science.1262110

Leung D, Jung I, Rajagopal N, Schmitt A, Selvaraj S, Lee AY, Yen CA, Lin S, Lin Y, Qiu Y, Xie W, Yue F, Hariharan M, Ray P, Kuan S, Edsall L, Yang H, Chi NC, Zhang MQ, Ecker JR, and Ren B. 2015. Integrative analysis of haplotype-resolved epigenomes across human tissues. *Nature* 518:350-354. 10.1038/nature14217

Ward LD, and Kellis M. 2012. HaploReg: a resource for exploring chromatin states, conservation, and regulatory motif alterations within sets of genetically linked variants. *Nucleic Acids Res* 40:D930-934. 10.1093/nar/gkr917

Wu C, Jin X, Tsueng G, Afrasiabi C, and Su AI. 2016. BioGPS: building your own mash-up of gene annotations and expression profiles. *Nucleic Acids Res* 44:D313-316. 10.1093/nar/gkv1104

Wu C, Orozco C, Boyer J, Leglise M, Goodale J, Batalov S, Hodge CL, Haase J, Janes J, Huss JW, 3rd, and Su AI. 2009. BioGPS: an extensible and customizable portal for querying and organizing gene annotation resources. *Genome Biol* 10:R130. 10.1186/gb-2009-10-11-r130
